# OpenCFU, a New Free and Open-Source Software to Count Cell Colonies and Other Circular Objects

**DOI:** 10.1371/journal.pone.0054072

**Published:** 2013-02-15

**Authors:** Quentin Geissmann

**Affiliations:** 1 Department of Animal and Plant Sciences, University of Sheffield, Sheffield, United Kingdom; 2 Institute for Biology, Free University of Berlin, Berlin, Germany; Centrum Wiskunde & Informatica (CWI) & Netherlands Institute for Systems Biology, The Netherlands

## Abstract

Counting circular objects such as cell colonies is an important source of information for biologists. Although this task is often time-consuming and subjective, it is still predominantly performed manually. The aim of the present work is to provide a new tool to enumerate circular objects from digital pictures and video streams. Here, I demonstrate that the created program, OpenCFU, is very robust, accurate and fast. In addition, it provides control over the processing parameters and is implemented in an intuitive and modern interface. OpenCFU is a cross-platform and open-source software freely available at http://opencfu.sourceforge.net.

## Introduction

Counting objects has always formed an important element of data collection in many fields of biology. It is therefore very common for biologists to enumerate objects such as pollen [1], eggs [2], seeds [3], nuclei [4], cells [5] or organisms [6]. Given that such tasks are time-consuming and, to some extent, subjective, it is surprising that automation is still infrequent. Effectively, enumerating objects is a two-part process: image capture and image analysis. Nowadays, technologies such as digital cameras and webcams provide an increasingly high image quality and are increasingly inexpensive. Simultaneously, many optimised image processing algorithms and open-source libraries can be used on laptops and desktop computers.

In different fields of microbiology, immunology and cellular biology, counting colonies of cells growing on agar plates is routine. Automating such counting procedures is not simple since colonies must first be isolated from the background and then, if they overlap, be separated. In addition, such methods must be capable of rejecting common artefacts such as imperfections in the agar, dust and edges of Petri dishes. However, since cell colonies are topologically fairly simple objects, solutions to enumerate them from pictures have long since been considered [7], [8]. Commercial tools have been developed [9], but remain expensive. Furthermore, the fact that the programs they provide are proprietary (*i.e*. not open-source) makes them very restrictive: it is rarely possible to know the precise nature of the analysis being performed, nor to modify or share these programs. In contrast, some authors have recently published open-source methods to count colonies from digital images [10]–[16]. These use a variety of techniques such as generalised Hough transform [11], template matching algorithm [5], advanced illumination correction and particle filter [15], watershed algorithm on a thresholded image [10], [14] and, more recently, a two-pass thresholding procedure in combination with statistical models [16]. They were either implemented as standalone programs capable of analysing image files [5], [11]–[13], extensions to existing image processing programs [10], [14], [15] or integrated (hardware-software) counting systems [16].

All these methods claim to give results very comparable to human counts, to improve objectivity and save time. However, none of them have been widely adopted. In addition to the fact that some biologists are not aware of the existence of such tools, their apparent failure to be adopted could be explained by shortcomings in performance, such as an inability to split merged colonies or long processing times. It is also very important for a method to be robust and versatile: it must be able to perform well without changing parameters when optimal conditions vary slightly, and the presence of artefacts should only have minor consequences. Another reason for non-adoption of automatic methods could be their lack of user-friendliness: even a perfect method may fail to be adopted if the software that implements it does not provide a modern user interface. Such an interface should enable the user to easily submit a list of images for analysis and to manually alter the values of the processing parameters. Additionally, the user should be able to select a region of interest and visually check the results. Since many users do not have deep knowledge of the underlying image processing, it is also important that the parameters are intuitive and few. A final reason could be the lack of public availability and maintenance. It is not rare that authors publish work about a program without mentioning a download link, releasing the software on a public repository or enclosing it as a supplementary material[5], [11]. In addition, a program will most likely need to be updated to correct unpredicted behaviour or avoid reliance on obsolete dependencies.

The aim of the work presented herein is to provide an alternative open-source tool that features very robust, accurate and fast image processing as well as a modern and functional user interface. Thanks to the optimised OpenCV library [17], a rapid implementation of image processing functions has been possible. This renders the analysis of very large and numerous picture-sets easy and offers integration of capture devices (such as webcams). In order to assess the relative efficacy and usefulness of the new software, a comparison to two other available tools was undertaken.

The NIST's Integrated Colony Enumerator (NICE) [13] software implements a combination of threshold and extended minima in order to improve robustness. The program comes with a functional user interface relying on few intuitive parameters and provides accurate results. However, it is relatively slow and lacks postprocessing filters. More recently, Cai *et al*. published [10] a short ImageJ macro which they described as very accurate and only depending on two parameters. The method relies on adaptive thresholding, watershed algorithm and subsequent particle filtering. It does not provide a functional user interface or indicate a way to open a list of files and display the outlines of the detected colonies, nor does it allow postprocessing of the detected colonies. Since ImageJ macro language is flexible, it is possible, but requires some familiarity with the language.

My results show that OpenCFU, the software created, is faster, more accurate and more robust to the presence of usual artefacts than NICE and Cai *et al*. 's macro. As well as efficiently counting bacterial colonies, the program can also be used to enumerate other circular objects such as seeds or pollen.

## Results

### Algorithm

**Figure 1:**
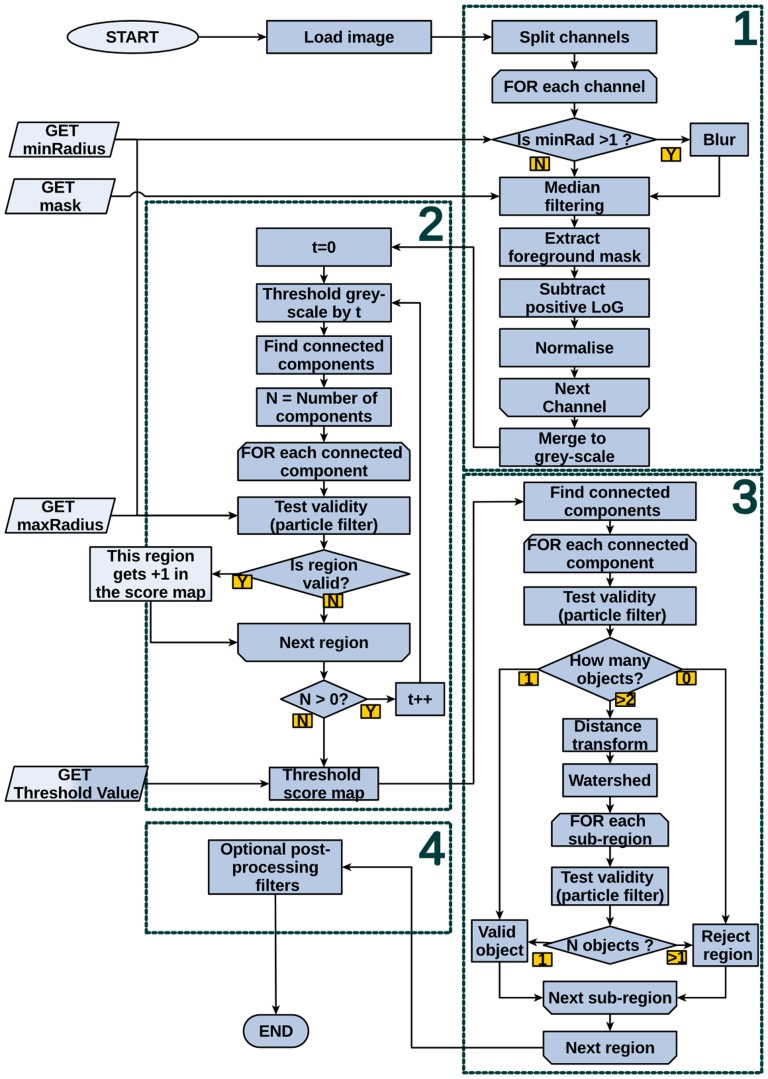
Flowchart representing the processing steps. The image is preprocessed (1) in order to correct for gradual changes in background intensity and increase the contrast. The first pass of the processing (2) generates a score-map by iteratively annotating valid regions. The second pass (3) involves finding connected components in the thresholded score-map and segmenting them using a distance transform/watershed approach. Optional postprocessing filters (4) can be performed by OpenCFU or, using the raw data, by the user.

**Figure 2:**
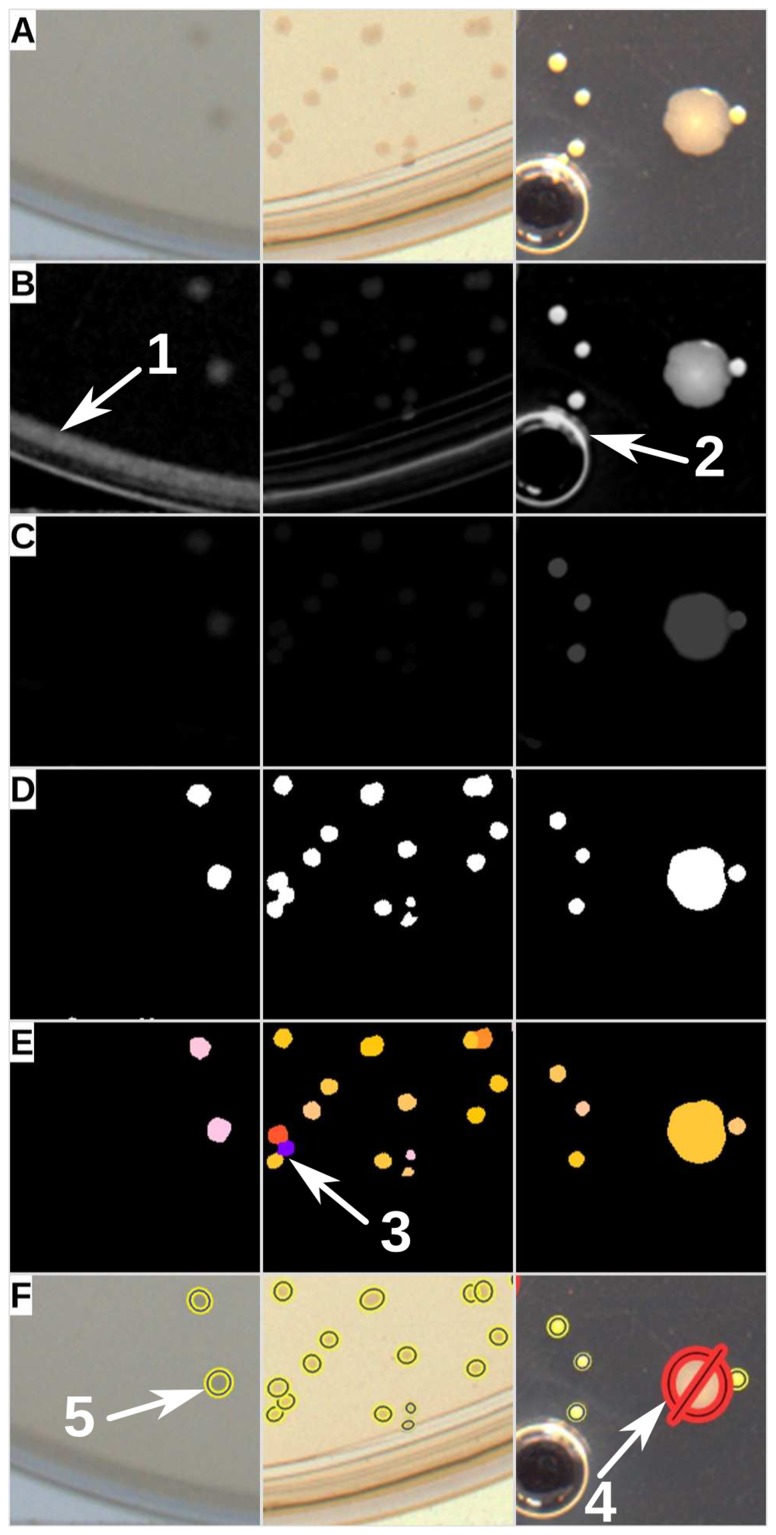
Illustration of the processing steps performed on three sample images. Each channel of the original image (A) is preprocessed individually and merged to a grey-scale image (B). A score-map is generated by recursive thresholding an annotation of circular regions (C). This excludes regions that were morphologically unlikely to be colonies (*i.e*. arrows 1 and 2). The score-map is then thresholded by a user-defined or automatically calculated value (D). The objects identified as merged colonies (on the basis of their morphological features) are segmented using a watershed variant on their distance transform (E). Arrow 3 shows objects that have been successfully segmented. Finally, the morphologically valid objects can be assessed further using intensity and colour filters. Arrow 4 shows a minority contaminant bacteria that was excluded using postprocessing filter and represented by crossed-out red ellipses. Arrow 5 shows valid colonies represented by yellow and blue ellipses. For the purpose of explanation, only representative areas (200
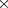
200 pixels) of three processed images are shown here.

Methods relying on direct thresholding of a grey-scale image followed by morphological segmentation are likely to detect high contrast artefacts such as parts of the edges of Petri dishes and bubbles. For a given value of threshold, some portions of these artefacts could, by chance, be morphologically similar to colonies and will result in detection of false positives. The algorithm proposed here aims to increase robustness by virtually testing all possible values of threshold and keeping only regions that were recurrently morphologically valid. A detailed flowchart is provided in figure 1 and portions of images at different processing stages are shown in figure 2. Briefly, the colour image is split into three channels from which the background is independently estimated using a local median filter. In order to enhance discrimination of foreground objects, the positive Laplacian of Gaussian of each channel's foreground mask is subtracted from itself and three preprocessed channels are normalised and merged to form a new grey-scale image.

The first pass of the processing involves thresholding the resulting grey-scale image by multiple values. For each value, the algorithm will search for connected components in the binary image. Each component is assessed by a particle filter that takes into account relationships between variables such as area, perimeter, convexity, aspect ratio and hollowness in order to determine whether or not a region is likely to be valid (*i.e* made from one or more circular objects). Over the range of threshold values, every time a valid region is found, all its pixels are incremented in a “score-map”. The score-map can be understood as a representation of how recurrently (over the iterations of threshold) pixels are part of a circular region. Finally, a user-defined (or automatic) threshold is applied to the score-map. During the second pass of the processing, a similar particle filter is applied but this time it classifies the connected components as “invalid”, “individual object” or “multiple objects”. “Individual objects” are accepted and “invalid” ones are rejected immediately, whilst “multiple objects” are morphologically segmented using a variant of the watershed algorithm on their distance-map [18]. Finally, all segmented objects are reassessed by the particle filter.

Optionally, a normal distribution is fitted to the relative colour intensities of objects that were not split (since they are less often falsely positive). This distribution then serves to compute the likelihood of each object to be valid. Finally, a simple likelihood user-controlled threshold is applied to exclude marginal objects.

### Implementation and User Interface

For performance reasons, OpenCFU was programmed in C++. The image processing was implemented using OpenCV framework [17] which offers highly optimised image processing functions. The time-consuming loops were optimised further for multi-core architecture using OpenMP library [19]. The graphical user interface was designed using GTKmm. These three libraries are open-source, cross platform and regularly maintained.

OpenCFU was designed in order to accelerate the calibration phase by having a fast processing time and by immediately displaying results after parameters have been changed. In addition, when a parameter is changed, OpenCFU dynamically restarts the analysis from the first step involving this variable, as opposed to systematically re-analysing the image from the beginning. For instance, if the value of a postprocessing filter is altered, the whole processing will not be re-run. The software also integrates an interface to video devices and other features such as optional automatic dish detection based on Hough circle transform.

The program can deliver two different types of result: a summary or a detailed output. In the summary, each row of data contains the name of the analysed image, the number of colonies detected in this image and, if a mask was used, the surface of the mask. In the detailed output, each row of data corresponds to a different colony. Each colony is characterised by the name of the image it comes from, the surface of the mask used for this image, the position (X, Y) of its centre, its corrected median values of colour intensity, its area, its perimeter and the number of colonies that were in the same cluster as this colony. This latter output is helpful for users needing to perform advanced analysis.

### Speed

**Figure 3:**
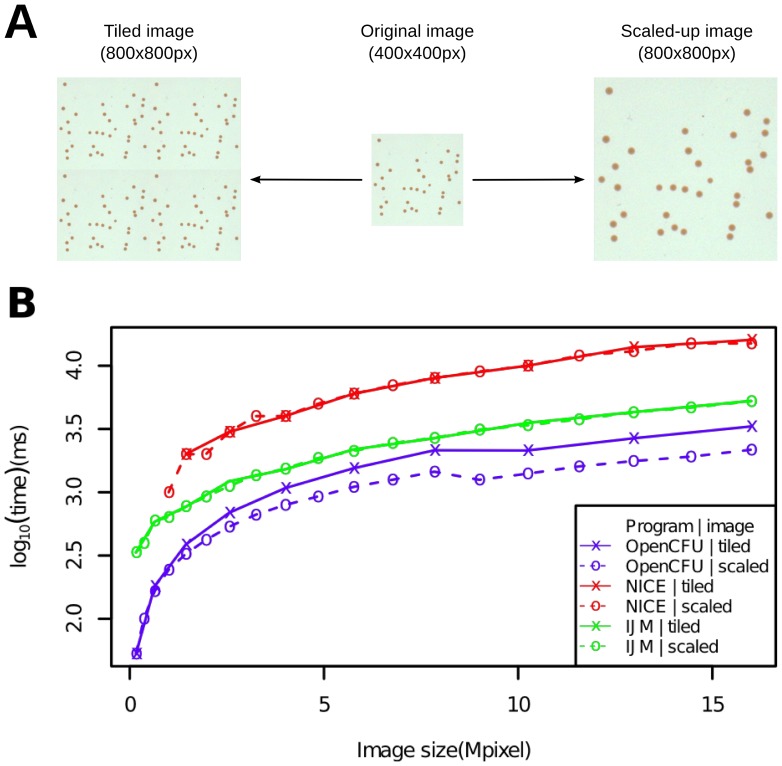
Processing time of OpenCFU, NICE [13] and an ImageJ macro [10] for images of different size. An original arbitrary square image was either tiled to itself or scaled-up (A) in order to obtain a range of square images featuring an increasing number of colonies or increasingly large colonies, respectively. The processing time of the three methods for these images was estimated in both cases (B). On the tested range of resolutions, OpenCFU was faster than both NICE and the ImageJ macro (IJM). The segments joining points do not represent data, but only aid readability.

Since algorithms are likely to iteratively process each of the foreground objects, their speed could differ according to their size and number. Therefore, in order to assess the method speed, two types of images were generated from the same template: “scaled-up” images featuring a constant number of increasingly big bacterial colonies, or “tiled” images with an increasing number of constant-sized colonies (fig. 3A).

The processing time for OpenCFU, NICE [13] and an ImageJ macro by Cai and colleagues [10] (IJM), were compared while analysing the same images (fig. 3B). Under the tested conditions, the three algorithms performed in linear time (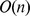). OpenCFU was the fastest, followed by IJM and NICE. For instance, for a typical picture of 1.6
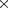
1.6kpx the tools would take approximatively 0.69, 1.22 and 3.0 seconds, respectively.

### Accuracy

In order to assess the accuracy of OpenCFU, 19 plates containing between 10 and 1000 *Staphylococcus aureus* colonies were prepared, independently enumerated by seven trained humans and then photographed with a high-definition camera. The pictures were analysed by OpenCFU, NICE and IJM. The results obtained by humans and automatic methods were then compared. The deviations of the results of each agent from the medians of human counts, the reference, were calculated.

**Figure 4:**
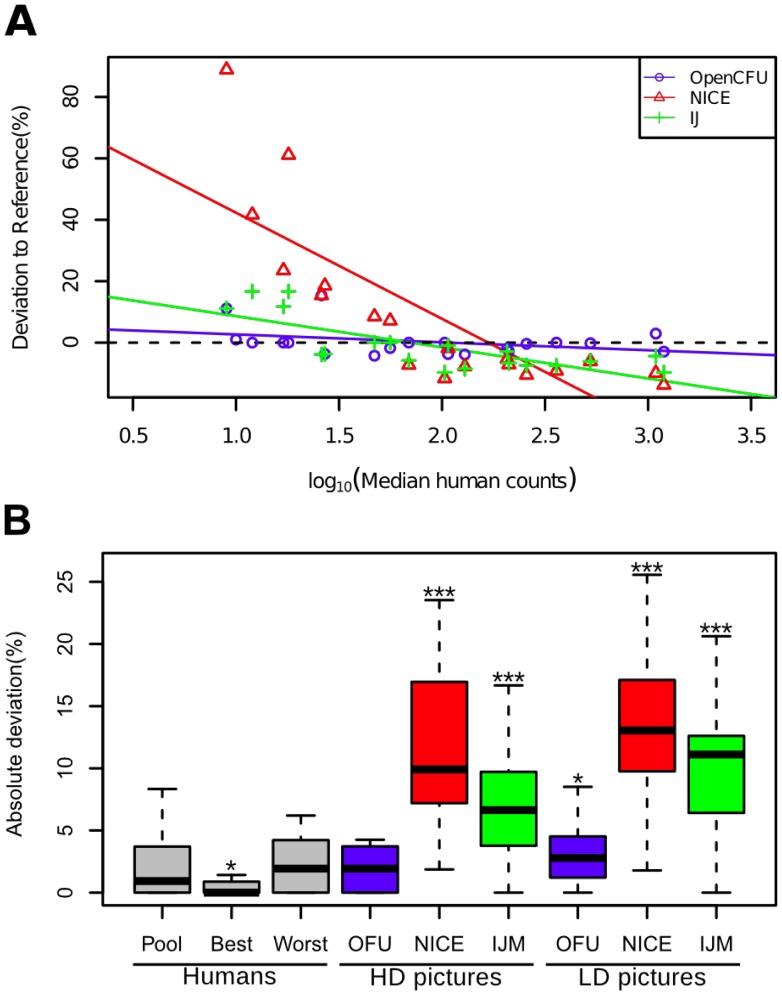
Comparison of accuracy between OpenCFU, NICE [13] and an ImageJ macro [10]. The medians of seven humans counts were used as a reference to measure deviation. The effect of the number of colonies on the deviation from the reference was assessed (A). For NICE and the ImageJ macro (IJM), the slope was significantly negative. The dotted line represents the reference. The absolute deviation from the reference was used as a measure of error (B). Error for the best human, the worst human and the three methods were compared to the pooled human group. With high-definition images (HD), NICE and IJM had a higher error than the pooled human group (Pool) while OpenCFU (OFU) did not. Using low-definition pictures (LD) from a low-cost webcam increased the error for the three methods.

NICE and, to a lesser extent, IJM tended to overestimate the number of colonies when few are present whilst underestimating the high-density plates (fig. 4A). The significance of the slopes of the linear regressions was assessed by a t-test: 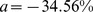 (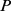-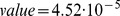) and 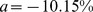 (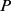-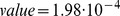), respectively. Analysis by OpenCFU did not result in a significant bias: 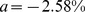 (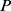-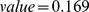).

The average of absolute deviations from the reference was used as a measure of error and compared between agents (fig. 4B). Images of the same plates were also taken with a low-cost webcam and analysed in order to estimate the impact of poor quality images on the accuracy of the three methods. The median error was 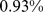 for human agents. In order to assess the inaccuracy of the automatic methods, their errors were compared to the human errors by performing a Wilcoxon test: NICE (median error 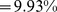) and IJM (median error 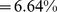) had a significantly higher inaccuracy than humans (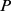-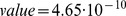 and 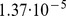, respectively). In comparison, OpenCFU (median error 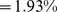) was not less accurate (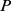-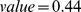). As expected, using poor quality pictures increased the error for OpenCFU (median error 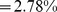; 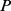-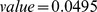), NICE (median error 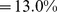; 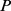-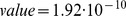) and IJM (median error 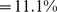; 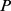-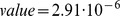).

### Robustness

**Figure 5:**
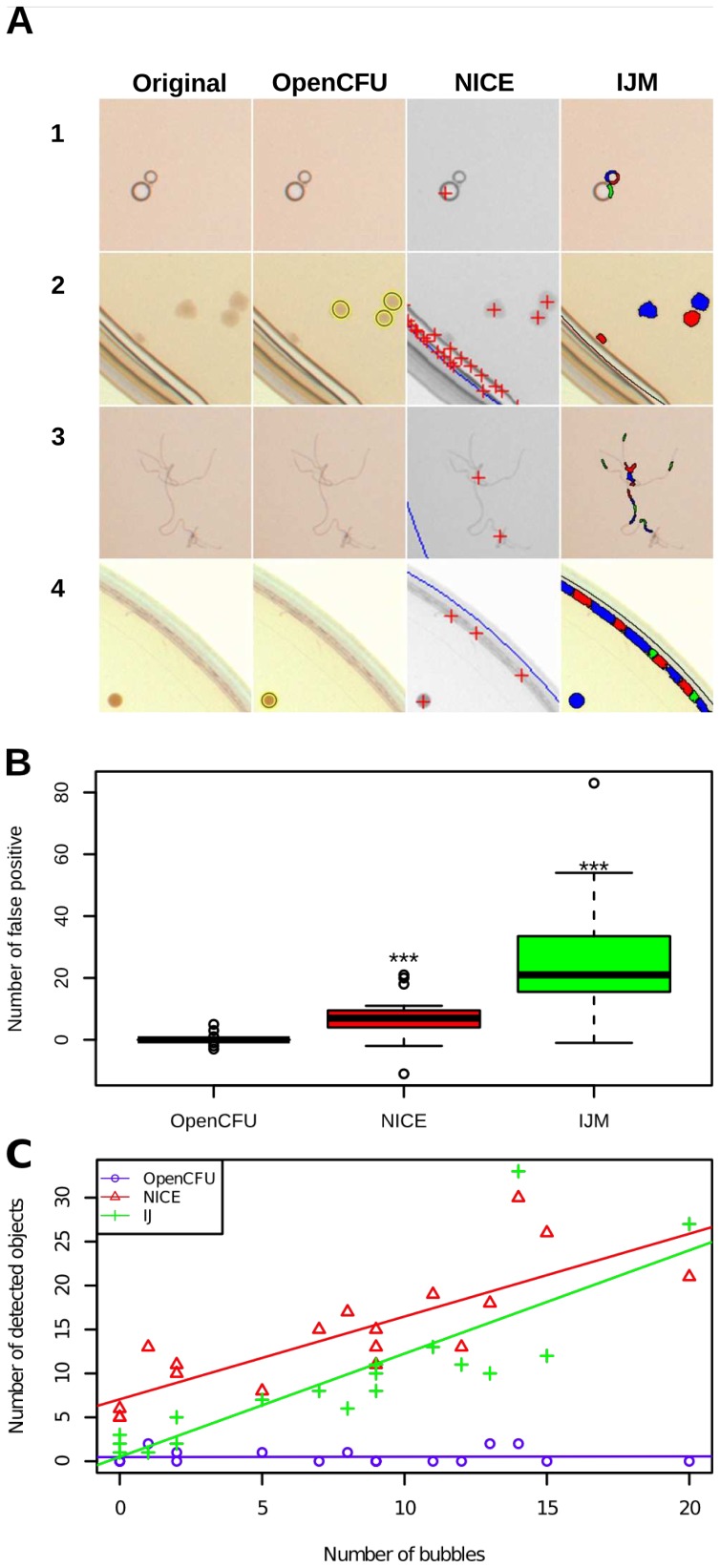
Comparison of robustness to common perturbations between OpenCFU, NICE [13] and an ImageJ macro [10]. A qualitative assessment of robustness was undertaken by analysing pictures containing artefacts (A). Representative portions of 1.7 cm by 1.7 cm (200
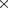
200 pixels) illustrate the results of the presence of bubbles (1), cracks in the agar (2), dust (3) and edge of dish (4) in the region of interest. Objects detected by OpenCFU, NICE and the ImageJ macro (IJM) are represented by ellipses, crosses and arbitrary colours, respectively. NICE and IJM but not OpenCFU seemed to consistently detect artefacts as colonies. A quantitative analysis of robustness to plate mispositioning was conducted (B). OpenCFU, NICE and IJM were used to count the number of colonies in the pictures of 19 plates. Then, all the images were translated by 1.7 mm (25px) and analysed with the same region of interest as the original. This procedure induced a significant bias for NICE, 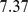 (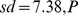-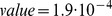) colonies and IJM 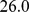 (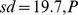-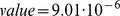) colonies, but not for OpenCFU 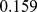 (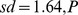-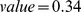) colonies (one-sided paired t-test). The impact of the presence of bubbles in the agar was measured by analysing pictures of 18 plates containing exclusively bubbles (C). A linear regression between the number of bubbles and the number of detected objects was performed. NICE and IJM counts were both positively related to the number of bubbles, 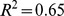 (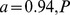-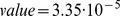) and 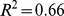 (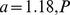-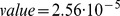), respectively. OpenCFU was not affected: 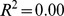 (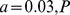-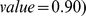.

In order to assess how robust the three methods were, pictures of plates featuring typical artefacts were analysed. Figure 5A shows qualitative results of this approach. Both NICE and IJM seemed likely to falsely count bubbles, edges, cracks or dust, whilst OpenCFU appeared unaffected.

To quantify the robustness of the three methods to the presence of edge, the images used for the accuracy test (fig. 4) were translated by 25 pixels to the top-left corner to simulate a slight (1.7 mm) mispositioning during acquisition (fig. 5B), and were re-analysed with the same parameters. The bias induced was assessed by measuring the difference between the results before and after perturbation. A paired t-test was performed between the original results of the three methods and their respective results after perturbations. In these conditions, IJM and NICE overestimated their own result by 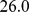 (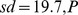-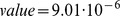) and 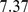 (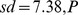-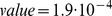) colonies, respectively. OpenCFU was not affected: 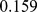 (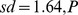-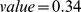) colonies.

**Figure 6:**
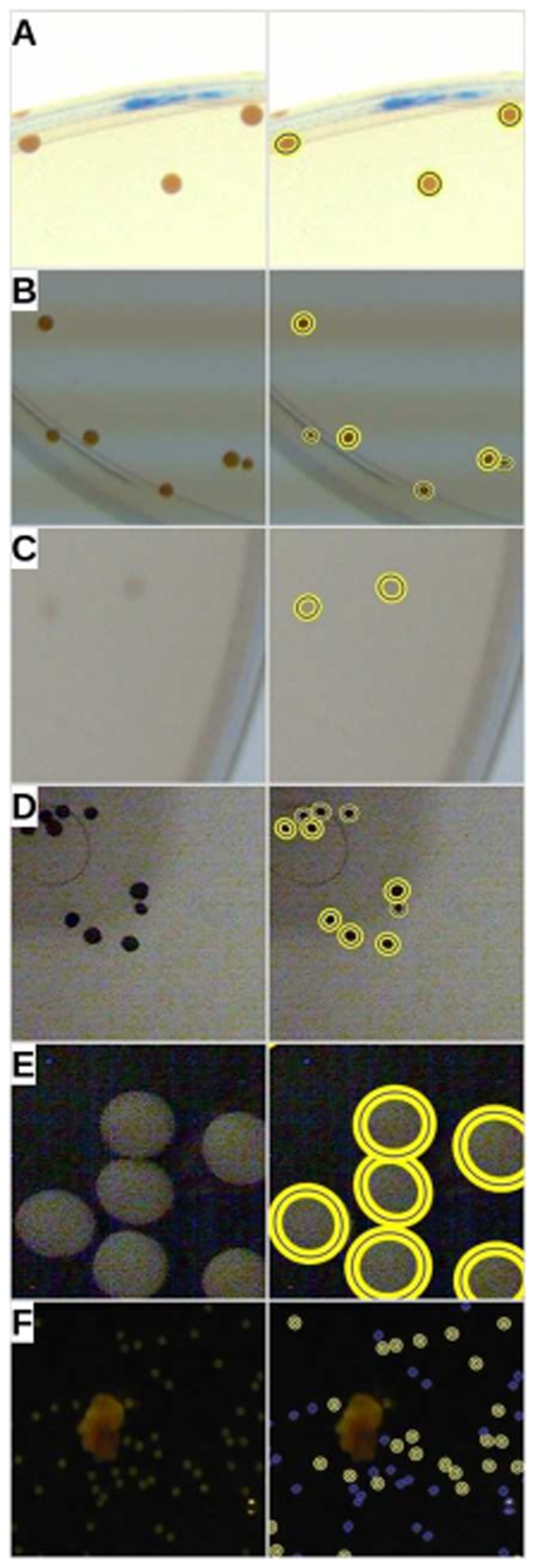
Versatility of OpenCFU. A qualitative assessment of the versatility of OpenCFU was undertaken by analysing pictures of different circular biological objects: a clear (A) and a poor quality (B) picture of *Staphylococcus aureus* colonies, a low-contrasted picture of *Escherichia coli* (C), a noisy picture of mustard seeds (D), a noisy picture of soy-bean seeds (E), and a micrography of *Carduus sp*. pollen (F). For the purpose of explanation, only representative areas (200
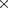
200 pixels) of six processed images are shown here. Original portions of images are on the left and correspond to the graphical results obtained using OpenCFU on the right.

In order to quantify the extent to which the presence of bubbles in the agar matrix would impact on the precision of the methods, 18 plates containing exclusively bubbles (between 0 and 20) were analysed. The relationships between number of bubbles and number of detected colonies is represented in figure 5C. Linear regressions were performed and the significance of the slopes was assessed by a t-test. The number of objects detected by IJM and NICE were positively related to the number of bubbles, 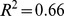 (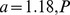-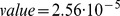) and 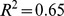 (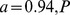-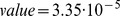), respectively. This was not the case for OpenCFU, 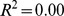 (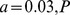-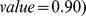. In order to qualitatively assess the ability of OpenCFU to process images of diverse nature, representative pictures of circular biological objects were analysed. Graphical outputs are shown in figure 6. These results suggest that the algorithm is versatile enough to enumerate circular objects from very different images.

## Discussion

In the present study, a new algorithm based on recursive research of circular regions over values of threshold of a grey-scale image has been presented, implemented and compared to two alternative methods [10], [13]. Comparison with additional software tools was considered, but, for different reasons, could not be formally performed. Among them, Clono-counter [12] can certainly be useful in some situations; however, it seems inappropriate for analysing numerous or large images. The program does not allow the user to analyse successively several files, and, for instance, using the same mask for different images is not possible. In addition, large images need to be down-scaled before analysis; as an objective comparison between programs requires all methods to process the same images, it was not possible to use Clono-counter. Both CHiTA [11] and Arraycount [5] have probably been very useful to some researchers in the past, but they were both unavailable for download and could not be compared to the present method. The method based on Cell profiler [15] could, in my case, not be adapted through minor alterations in the proposed pipeline. The ImageJ plug-in developed by Sieuwerts *et al*. [14] involved several human interventions (converting to grey-scale and thresholding). In addition, a general, rather than adaptive, threshold on the grey-scale image was, in my case, not satisfying since background intensity varied between and within plates. For this reason, this plug-in was not included either. Finally, the results presented by S. Brugger and co-workers [16] are promising, but they proposed a colony counter relying on a specific acquisition platform rather than a standalone program capable of processing a range of pictures from different devices.

Despite the intensive nature of the image processing, the implementation of the described algorithm has been shown to perform even faster than methods that are supposedly less intensive [10], [13] (fig. 3). This is most likely due to the use of the optimised OpenCV library [17] in combination with custom C++ functions. For a standard picture, the three tested methods performed in less than three seconds. In comparison, much slower methods, such as the one presented by Vokes and Carpenter [15], would take approximately two minutes for the same image (data not shown). Since, on modern hardware, the processing time will rarely be much longer than the total acquisition time, OpenCFU's faster performance is unlikely to provide a decisive direct gain of time over NICE [13] and the ImageJ macro published by Cai and colleagues (IJM) [10]. However, authors have acknowledged that calibration is the most time-consuming step of semi-automatic methods [4], [19]. Since, during calibration, a human will essentially try multiple values of parameters on different sample images, a fast processing speed coupled with an immediate display of the result will certainly provide an advantage. In addition, OpenCFU will save calibration time by only reprocessing an image from the first step involving the parameter that has been changed rather than from the beginning.

OpenCFU was shown to be very accurate and, with high-definition pictures, did not generate more errors than the average human error (fig. 4). In contrast, NICE and IJM were shown to overestimate plates with few (less than 50) colonies and underestimate plates with large numbers of colonies (fig. 4A). They also had a significantly higher inaccuracy than humans (fig. 4B). NICE and IJM had to be used with regions of interest drawn inside the dish. This practice excludes colonies from the analysis and could result in a consistent underestimation. But such bias could also be a consequence of an inefficiency to detect or segregate small colonies. The overestimation of low-load plates was however probably the result of false positives arising from the presence of artefacts which are pragmatically hard to avoid. Using NICE with Otsu's method for thresholding gave overall good results (fig. 4A). However, it assumes foreground objects exist and would be inappropriate if only few (or no) colonies are present. One solution could be to manually count plates that have few colonies and use an image processing-based method for high-load plates. This will surely result in logistic complications and errors. Another solution is to individually verify each processed image and reprocess some with a different threshold. This is not easy with NICE because the user has to specifically click on image names one-by-one and then click each time to query a display of the colonies. Finally, if an image is judged to be misprocessed, there is no obvious manner to reprocess it exclusively. IJM does not provide a way to load a list of images and overlay results on top of the original images for visual verification. Achieving this is possible but involves some knowledge of ImageJ macro language.

Authors have emphasised the need to have a low-cost platform to count colonies and have, for instance, used desktop-scanners [9], [10], [13], [18] that are generally rather slow. In this study, a low-cost webcam used in combination with OpenCFU provided very satisfying results (fig. 4B). OpenCFU integrates video devices such as webcams, USB microscopes or firewire cameras in order to facilitate this approach.

For most biologists, a moderate average deviation (lower than 20%) will often be negligible compared to noise generated by other experimental factors. Rather than trying to provide a perfect similarity to human counts, methods based on digital image processing should focus on robustness and safety. In this study, OpenCFU has been shown to be very robust to the presence of artefacts in comparison to alternative methods (fig. 5A). A simulation of a small, but likely, physical perturbation of the settings – mispositioning the dish during acquisition – was proven to lead to a large overestimation of the number of colonies by alternative methods (fig. 5B). Even if a method is very accurate in perfect conditions, a biologist may be reluctant to use it if moving the acquisition set-up by less than two millimetres could add more than twenty colonies to the result. In order to limit the impact of such an eventuality, the experimenter will need to systematically check and sometimes reprocess images. Vokes and Carpenter [15] solved this problem by using a template mask and computing its best alignment to the actual image. As OpenCFU is, by design, very robust to the presence of edges, the use of a mask is rarely needed. In fact, the regions of interest defined in this study completely included the outside boundaries of the dishes. In addition, the software provides automatic detection of a Petri dish in the images which corrects for shifts of the dish. This feature also avoids the subjectivity of manually drawing a mask and could indirectly save acquisition time since the user can afford to take less care in positioning the dishes.

Unlike alternatives, OpenCFU was not affected by common artefacts such as bubbles in the agar matrix (fig. 5C). *Staphylococcus aureus* colonies, used in this study, are opaque and therefore produced well contrasted areas. As NICE and IJM do not perform a stringent morphological analysis of detected objects, their sensitivity to artefacts can be suspected to be even higher when analysing less contrasted objects. Although it is possible to verify that no artefacts are present and to redraw the mask if necessary, this step would be an additional subjective and time-consuming human intervention. Another advantage resulting from the robustness of the algorithm is the ability of OpenCFU to process a diverse range of images. Preliminary results (fig. 6) indicate that the algorithm is capable of enumerating different types of colonies, as well as objects such as round seeds and pollen from very different pictures.

In this study, humans took 39.3 (sd = 6.1) minutes in order to count the 19 plates. Theoretically, the three methods could achieve the same result in less than five minutes (including acquisition time). However, the lack of robustness of NICE and IJM could require systematic verification and rectification of the results after processing. The user interface of NICE does not make this task easy and IJM does not natively provide this function. Such flaws could negate the time-gain they would offer in perfect conditions.

OpenCFU provides postprocessing filters to eliminate detected objects according to their intensity and colour similarity. This makes the method even more robust since it can be used to exclude circular artefacts such as contaminant bacteria. Most frequently, biologists will write in a small area of the dish or in the edge; these filters can also be used to remove all writing, as it often has a very specific colour. OpenCFU can also produce detailed output containing information about each detected colony. This is useful for users who, for instance, wish to calculate the respective number of distinct populations of cells.

In this study, a new algorithm was used to count bacterial colonies and implemented in a modern and functional interface. In the tested conditions, OpenCFU has been shown to be faster, more accurate, and more robust to common perturbations than the two tested alternatives. OpenCFU will help to save time and reduce subjectivity of colony counting. Since many other biological objects (for instance, seeds, pollen, cells, nuclei and eggs) are circular and well defined from the background, they can also be counted without any modification of the software.

### Availability and Future Directions

OpenCFU is an open-source program distributed under the conditions of the GNU General Public Licence version 3. It is available on Sourceforge (http://opencfu.sourceforge.net). A user manual and video tutorial are also provided. In addition, an increasingly large list of image samples and corresponding results will be maintained in order to help users calibrate the method. The program will be improved and updated as users request features and bugs are pointed out. Planned improvements of the program include support for multiple regions of interest, user-supervised exclusion of outlier colonies and the availability of a command line version.

## Materials and Methods

### Image Processing

Details of the processing pipeline and its implementation can be freely viewed, modified and redistributed from the source code. The file “robustCircularRegions.cpp” implements the new algorithm. The particle filter and custom watershed function are in “metaContourFilter.cpp”. All the processing functions used are either directly provided by OpenCV or written in C++ (using OpenCV framework) in the same file.

The particle filter first rejects objects that do not fulfil all of the following conditions:
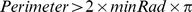


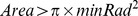


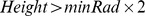
Where,


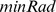
 is the user-defined minimal radius.

Then, it decides if a region is a single object (colony) or multiple clustered objects. To be a single object, it must fulfil at least one of the following criteria:
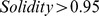


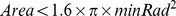


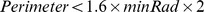
Where,



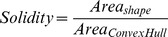



Otherwise, it is decided to be multiple objects.

If it is a single object, it must fulfil all of the following criteria:
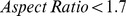


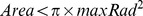


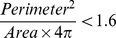
Where,


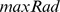
 is the user-defined (or calculated from the image dimensions) maximal radius for an object.

If it is a cluster of objects, it must fulfil all of the following criteria:
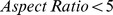


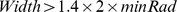


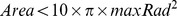


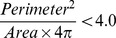



To segregate clustered objects – during the second pass of the processing – a distance transform is computed on a 5
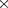
5 pixel neighbourhood with a = 1, b = 1.4, c = 2.1969. The peaks of the distance transform serve as markers for a custom watershed function. Briefly, the watershed-like function works as follows:

All markers correspond to the local maxima in the distance-map.

As long as marked regions can grow:

If a marked pixel has a higher or equal value than a neighbour and the neighbour is not marked, the neighbour becomes marked with the same label.

In addition, marked regions are not allowed to expand their area over a limit value 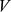:
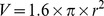



And the distance between the original marker of a region and any pixel of this region must be lower than 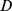:
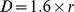
Where,


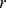
 is the value of the corresponding peak in the distance-map (*i.e.* the presumptive radius).

### Plates Preparation and Manual Counting

LB broth supplemented with 1.5% agar was poured into 19 standard 90 mm plates. An overnight culture of *Staphylococcus aureus* was diluted and 100
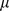
L were plated. The bacterial solution was spread using ten 2 mm glass beads. The plated bacteria were grown overnight at 37°. Seven trained individuals were given the 19 plates in a random order. The experiment was blinded so that no subject could know the results of any other before counting. The total time they took (excluding copying data to an electronic file) was recorded. Plates with bubbles (fig. 5C) were obtained by injecting between 0.5 and 1
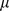
L of air in the agar matrix. Sometimes, bubbles were surrounded by one or two smaller adjacent bubbles. Under this scenario, only the largest was counted.

### Semi-Automatic Methods

OpenCFU version 3.3 was used with a “threshold” value of 12 for high-definition images and 7 for webcam images. The mask was drawn automatically for all plates by choosing the option “Auto-Petri” with a margin of −25px. The software NICE and the necessary proprietary MATLAB Complier Runtime were both downloaded from http://www.nist.gov/pml/div682/grp01/nice.cfm. An elliptic region of interest was manually drawn and applied for all plates in order to exclude the edge of the Petri dishes and the Otsu thresholding method was used with the “high resolution” setting for high-definition pictures and “medium resolution” for the webcam images. The ImageJ macro was adapted from Cai's publication [10] with minor modifications. The threshold (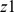) was 205 and the minimal size (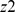) was 15.

### Hardware

The high quality pictures were taken using QIMAGING Micropublisher 3.3RTV device with a TAMRON 1∶1.4 25 mm ø30.5 lens. The pictures generated were 1536
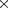
1538px well-contrasted images. The webcam used as a capture device for the real-time enumeration was a Sweex Blackberry Black WC250 (1600
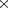
1200px, 30fps). A white trans-illuminator was used to optimise contrast in both cases.

The processing time of OpenCFU, NICE and IJM were assessed on the same machine: a “System76 Gazelle Professional” (CPU  =  intel i7-2630QM, 2.00GHz). OpenCFU and IJM were used under GNU/Linux operating sytem (Linux Mint 13, kernel 3.2). OpenCFU was compiled with g++4.6.2 and OpenCV 2.4.2. IJM was used with ImageJ 1.46 (Java 1.6.0_24). NICE was used under Windows7-professional (64bit).

### Statistical analysis

In order to assess the effect of the number of colonies on the deviation from the reference (fig. 4A), a linear model between deviation and 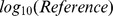, was fitted. A t-test was performed on the slope of the regression line.

The deviation in the count of each plate was given by:
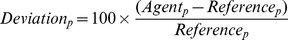
Where,


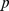
 is the plate and 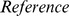 the median of human counts for 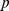.

The absolute deviations from the reference (fig. 4B) were compared between each agent and the pooled human group by performing a Wilcoxon test. In order to assess the significance of the greater number of detected colonies after translation of images (fig. 5B), a one-tailed paired t-test comparing the numbers before and after perturbation was performed. In order to quantify the effect of the number of bubbles on the number of detected colonies (fig. 5C), a linear model was fitted and a t-test was performed on the slope of the regression line. Statistical analysis was performed using R software [20].
